# A Review of Risk Scores within Upper Gastrointestinal Bleeding

**DOI:** 10.3390/jcm12113678

**Published:** 2023-05-26

**Authors:** Josh Orpen-Palmer, Adrian J. Stanley

**Affiliations:** Department of Gastroenterology, Glasgow Royal Infirmary, Glasgow G4 0SF, UK

**Keywords:** acute gastrointestinal haemorrhage, risk stratification, risk scores, non-variceal upper gastrointestinal bleeding, variceal gastrointestinal bleeding, peptic ulcer bleeding

## Abstract

Upper gastrointestinal bleeding is a common medical emergency. Thorough initial assessment and appropriate resuscitation are essential to stabilise the patient. Risk scores provide an important tool to discriminate between lower- and higher-risk patients. Very low-risk patients can be safely discharged for out-patient management, while higher-risk patients can receive appropriate in-patient care. The Glasgow Blatchford Score, with a score of 0–1, performs best in the identification of very low-risk patients who will not require hospital based intervention or die, and is recommended by most guidelines to facilitate safe out-patient management. The performance of risk scores in the identification of specific adverse events to define high-risk patients is less accurate, with no individual score performing consistently well. Ongoing developments in the use of machine learning models and artificial intelligence in predicting poor outcomes in UGIB appear promising and will likely form the basis of dynamic risk assessment in the future.

## 1. Introduction

Upper gastrointestinal bleeding (UGIB) is defined as bleeding proximal to the ligament of Treitz. It is a common medical emergency with an annual incidence rate of up to 172 per 100,000 people [1]. Despite advancements in management with improved pharmacological and endotherapeutic intervention, mortality remains approximately 10% in the context of an increasingly elderly, comorbid population [2]. Stratification of patient using validated risk scores has become increasingly important to clinicians, enabling identification of those at risk of poor outcomes and directing appropriate management and level of care.

Multiple risk scores for UGIB have been developed, to predict outcomes including mortality, requirement for endoscopic or other haemostatic intervention and risk of rebleeding. More recently, with increasing pressures on healthcare systems globally, identification of very low-risk patient who can be safely discharged early for out-patient management has increased in priority. No single risk score has been shown to be accurate at measuring all relevant outcomes. This article will review the strength and weakness of these scoring systems and the potential for future use and development.

## 2. Risk Scores

UGIB risk scores can be broadly classified in two major groups: pre-endoscopic and post-endoscopic. Post-endoscopic risk scores include the complete Rockall (RS), Progretto Nazional Emoragia Digestiva (PNED) and Cedars Sinai Medical Centre Predictive Index (CSMCPI). Pre-endoscopic risk scores do not depend on visualisation of the GI tract, enabling calculation soon after admission which has clinical advantages [3]. Pre-endoscopic scores include the pre-endoscopy Rockall (pRS), Glasgow Blatchford (GBS), AIMS-65 (albumin, international normalised ratio, mental status, systolic blood pressure, age ≥65 yrs,), ABC (age, blood tests, co-morbidities), Canada–United Kingdom–Adelaide (CANUKA) and MAP (ASH) (mental status, American Society of Anaesthesiology (ASA) classification, pulse, albumin, systolic blood pressure, haemoglobin) score. In addition to these pre- and post-endoscopic scores, the Forrest classification of ulcer morphology used by endoscopists in UGIB can predict risk based on endoscopic parameters in isolation. 

These scores were developed to predict different primary endpoints and have subsequently been compared against one another in numerous studies. The GBS, AIMS-65 and pRS have been most widely externally validated [4].

Risk scores are usually evaluated by determining the area under the receiver operating characteristic curve (AUROC). An AUROC of 0.5 indicates chance, while a score of 1.0 indicates absolute sensitivity and specificity. A score of >0.9 indicates excellent performance, while a score >0.8 is generally considered good and clinically meaningful [5].

### 2.1. Pre-Endoscopy Risk Scores

#### 2.1.1. Glasgow Blatchford Score

The GBS was developed and published in 2000 and was designed to predict need for in-patient treatment (defined as blood transfusion, endoscopic therapy or surgery. Based on 1748 patients and scored in a range of 0–23, it is composed of seven variables: haemoglobin, urea, heart rate, systolic blood pressure, co-morbidity and presenting symptoms (syncope and melaena) [6]. It was externally validated across four UK sites in 2009 and has also been widely studied internationally [7,8].

**Table 1:** The Glasgow Blatchford Score.

Variable	Score
Heart Rate (beats per minute)	1
≥100	
Systolic Blood Pressure (mm Hg)	
100–109	1
90–99	2
<90	3
Blood Urea Nitrogen (mmol/L)	
6.5–7.9	2
8.0–9.9	3
10.0–24.9	4
>25.0	6
Haemoglobin (g/L)	
Male 120–130	1
100–119	3
<100	6
Female 100–120	1
<100	6
Other Markers	
Presentation with melaena	1
Presentation with syncope	2
Hepatic Disease	2
Cardiac Failure	2

#### 2.1.2. AIMS-65

Published in 2011, this American score was based on 29,222 cases from a US-wide database between 2004 and 2005. The score was subsequently validated by the authors using data from the same database between 2005 and 2006 based on 32,504 cases. Primary outcomes were mortality, LOS and cost of admission. The five variables noted to have best discrimination and therefore adopted were albumin (A), international normalised ratio (I), altered mental status (M), systolic blood pressure (S) and age ≥65 yrs [9].

**Table 2:** The AIMS-65 Score.

Variable	Score
Albumin <30 g/L	1
International Normalised Ratio >1.5	1
Altered Mental Status	1
(Glasgow Blatchford Score <14, Lethargy, Stupor or Coma)	
Systolic Blood Pressure (mm Hg) ≤90	1
Age >65 years	1

#### 2.1.3. Age, Blood Test, Co-Morbidities (ABC)

The ABC is a more recent score published in 2020 with 30-day mortality as the primary outcome, designed for utilisation in both UGIB and lower gastrointestinal bleeding (LGIB). In total, 3012 UGIB patients were included in the development cohort from numerous international sites with subsequent validation using 4019 UGIB and 2336 LGIB patients. The ABC uses six variables: age, blood tests (urea, albumin, creatinine), and co- morbidity including ASA classification [10].

**Table 3:** The ABC score.

Variable	Score
Age	
60–74 years	1
≥75 years	2
Blood tests	
Urea >10 mmol/L	1
Albumin <30 g/L	1
Creatinine (µmol/L)	
100–150	1
>150	2
Co-morbidity	
Altered Mental Status	2
Liver Cirrhosis	2
Disseminated Malignancy	4
ASA Classification	
3	1
≥4	3

#### 2.1.4. MAP(ASH)

Published in 2019, the MAP(ASH) included 547 patients in the development cohort and subsequently 3012 patients in the validation cohort. The six factors included in the score are mental status (M), ASA classification (A), pulse (P), albumin (A), systolic blood pressure (S) and haemoglobin (H) [11].

**Table 4:** The MAP (ASH) score.

Variable	Score
Altered Mental Status	1
(Glasgow Blatchford Score <15)	
ASA Classification >2	1
Heart Rate (beats per minute) >100	1
Albumin <25 g/L	2
Systolic Blood Pressure (mm Hg) ≤90	2
Haemaglobin (g/L) <10	2

#### 2.1.5. Canada–United Kingdom–Adelaide

Another relatively recent risk score, the CANUKA was published in 2018. Developed through multivariable logistic regression modelling of 10,639 cases pooled from across Canada, the UK and Australia, it is designed to predict a composite endpoint of 30-day mortality, rebleeding, and requirement of intervention or red blood cell transfusion. It consists of nine parameters: age, presenting features (melaena, haematemesis, syncope), haemoglobin, urea, co-morbidity, heart rate and systolic blood pressure [12]. 

**Table 5:** The CANUKA score.

Variable	Score
Age	
50–64	1
≥65	2
Presenting Feature	
Melaena	1
Haematemesis	1
Syncope	1
Haemoglobin (g/L)	
10.1–12	1
8.1–10	2
≤8	3
Blood Urea Nitrogen (mmol/L)	
5–9.9	1
10–14.9	2
≥15	3
Co-morbidity	
Liver Disease	2
Malignancy	2
Heart Rate (beats per minute)	
100–124	1
≥125	2
Systolic Blood Pressure (mm Hg)	
100–119	1
80–99	2
<80	3

### 2.2. Post-Endoscopy Risk Scores

#### 2.2.1. Rockall Score

Published in 1996 and based on 4185 cases of UGIB, the Rockall score was designed to predict mortality. The full score ranges from 0 to 11 and includes five variables, two of which are dependent on endoscopy findings: age, haemodynamic stability, co-morbidities, endoscopic diagnosis, and stigmata of recent haemorrhage. The pRS excludes endoscopic findings (score 0–7) facilitating earlier use [13].

**Table 6:** The Rockall Score.

Variable	Score
Age	
60–79 years	1
>80 years	2
Shock Index	
Heart Rate (beats per minute) >100 and	
Systolic Blood Pressure (mm Hg) >100	1
Heart Rate (beats per minute) >100 and	
Systolic Blood Pressure (mm Hg) <100	2
Co-morbidity	
Cardiac failure, Ischaemic Heart Disease, Any Major Co-morbidity	2
Renal Failure, Liver Failure, Disseminated Malignancy	3
Endoscopic Diagnosis	
Mallory Weiss Tear, No Lesion	0
All Other Diagnoses	1
Malignancy of the Upper GI Tract	2
Stigmata of Recent Haemorrhage	
None, Dark Spot only	0
Blood, Adherent Clot, Spurting vessel	2

#### 2.2.2. Cedars Sinai Medical Centre Predictive Index

This was developed in the same year as the Rockall score, with an initial study size of 500 patients, and the CSMCPI is also scored in a range of 0–11. It was designed to predict two main endpoints, patient safety, measured by complication rate and hospital length of stay (LOS). It uses four variables: haemodynamic stability, co-morbidity, time from symptoms to hospitalisation, and endoscopic findings [14].

**Table 7:** The CSMCPI score.

Variable	Score
Haemodynamic Stability	
Stable	0
Intermediate	1
Unstable	2
Co-morbidity	
2	1
3	2
≥4	3
Time from Onset of Symptoms to Hospitalisation	
<48 h	1
In hospital	2
Endoscopic Findings	
Ulcer without Stigmata of Recent Haemorrhage, Non Bleeding Mallory Weiss Tear	0
Ulcer with Flat Spot or Clot, Erosive Disease with Stigmata of Recent Haemorrhage, Angiodysplasia	1
Ulcer with Non-bleeding Vessel or Stigmata of Recent Haemorrhage	2
Persistent haemorrhage, Varices or Malignancy of the Upper GI Tract	3

#### 2.2.3. Progretto Nazional Emoragia Digestiva

Described in 2010, this Italian score utilised a national database involving 1360 patients. Scored in a range of 0–23, its primary outcome was 30-day mortality. Categorising patients as low, medium or high risk, it has seven components: age, time to admission, haemoglobin, co-morbidity, ASA classification, recurrent bleeding and failure of endoscopic treatment [15].

**Table 8:** The PNED score.

Variable	Score
Age ≥80 years	2
Time from Onset of Symptoms to Hospitalisation <8 h	1
Haemoglobin (g/L) ≤7	2
Co-morbidity	
Renal Failure	2
Neoplasia	3
Liver Cirrhosis	3
ASA Classification	
3	1
4	3
Rebleeding	3
Failure of endoscopic treatment	4

### 2.3. Endoscopic Risk Scores

#### Forrest Classification of Ulcer Stigmata

Developed in 1974, the Forrest classification has been widely adopted for description of ulcers at endoscopy. Six categories exist based on morphology: spurting or oozing lesions (Forrest Ia and Ib), non-bleeding visible vessels, adherent clots (Forrest IIa and IIb) and lesions with pigmented or clean base (IIc and III) [16]. In addition to providing a descriptive tool, this classification enables the classification of high- or low-risk ulcers, with Forrest Ia and Ib being independent risk factors for persistent or recurrent bleeding [17]. 

**Table 9:** The Forrest Classification of Ulcer Stigmata.

Lesion Characteristic	Ulcer Classification
Spurting bleed	Ia
Oozing bleed	Ib
Non Bleeding, visible vessel	IIa
Adherent Clot	IIb
Pigmented base	IIc
Clean base	III

## 3. ‘High-Risk’ Patients

The definition of high-risk patients with UGIB varies between studies but generally includes those who reach some or all of the endpoints: requirement of blood transfusion, endo-therapeutic, surgical or radiological intervention to achieve haemostasis, rebleeding and mortality. Early identification can help direct such patients to appropriate medical intervention, level of care and potentially optimise timing of endoscopy. The ideal risk score would accurately predict all these outcomes; however, no such score currently exists.

### 3.1. Predicting Mortality

Historically, mortality has been the main outcome of interest in UGIB, usually measured at 30 days. It is the primary outcome predicted by many UGIB risk scores including the Rockall, PNED, AIMS-65 and ABC. An international multicentre prospective study involving 3012 patients comparing the RS, pRS, AIMS-65, GBS and PNED found that the AIMS-65 (AUROC 0.77) was comparable to the PNED (AUROC 0.77) and was the best of the pre-endoscopic scores considered. GBS (AUROC 0.64, *p* < 0.001) and pRS (AUROC 0.72, *p* = 0.05) were inferior at predicting 30-day mortality [7]. This study also showed the optimal score thresholds for predicting mortality were PNED ≥ 4, AIMS-65 ≥ 2 pRS ≥ 4, RS ≥ 5, GBS ≥ 5.

The recently described ABC score was shown to be superior to AIMS 65 at predicting 30-day mortality in the initial derivation and validation publication including 4019 UGIB patients in the validation cohort (derivation cohort AUROC 0.86 vs. validation 0.81 vs. AIMS-65 0.65 *p* < 0.001) [10].

A single-centre Australian study involving 424 patients also suggested the AIMS-65 was superior to the GBS and pRS in predicting in-patient mortality (AUROC 0.8 vs. 0.76, *p* = 0.03 and 0.74, *p* = 0.001, respectively) [18]. However, a European series including 309 patients comparing the AIMS-65, GBS and RS reported no difference in the prediction of in-patient mortality (AUROC 0.76 vs. 0.78 vs. 0.78, respectively), although it suggested the AIMS-65 may more accurately predict delayed (six months) mortality (AUROC 0.74 vs. 0.66 vs. 0.71, respectively, *p* < 0.04) [19].

**Figure 1:**
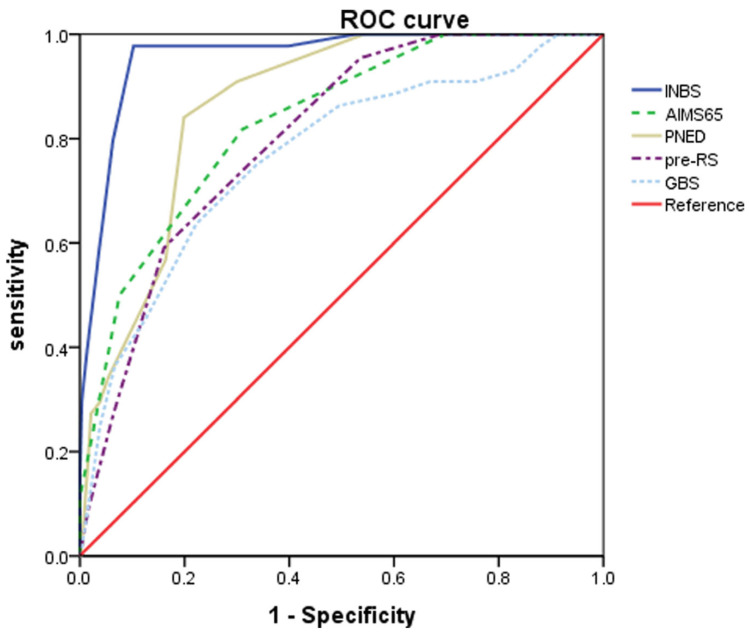
Comparison of scoring systems in the prediction of 30-day mortality *(n* = 905). AUROC, area under receiver operating characteristic curve (95% CI); INBS, international new bleeding score; Pre-RS, pre-endoscopic Rockall score; GBS, Glasgow Blatchford score; PNED, Progetto Nazionale Emorragia Digestiva score. Note the INBS was subsequently renamed the ABC score by the authors [20].

More recently, a large single-centre Korean retrospective study including 905 patients with non-variceal bleeding reported that the ABC score (AUROC 0.958, 95% confidence interval 0.943–0.970) was superior in predicting 30-day mortality compared to the AIMS-65 (AUROC 0.832, *p* < 0.001), PNED (AUROC 0.865, *p* < 0.001), pRS (AUROC 0.802, *p* < 0.001 and GBS (AUROC 0.765, *p* < 0.001) (Figure 1) [20]. A smaller single-centre cohort study of 310 patients also suggested the ABC score (AUROC 0.79) was superior to the AIMS-65 (AUROC 0.67 *p* < 0.001) and RS (AUROC 0.62, *p* < 0.001) at predicting 30-day mortality [21].

During internal validation, the authors of the CANUKA score found it had similar accuracy to GBS AUROC (0.77 vs. 0.74, *p* = 0.47) and pRS (0.77 vs. 0.79 *p* = 0.43) [12] in the prediction of mortality. External validation of the CANUKA is required.

Overall, the ABC score appears to be better than other pre-endoscopic established scores at predicting mortality, with reported AUROC figures consistently >0.8. The AIMS65 and CANUKA scores also appear to be promising but further comparison studies are required in different populations.

### 3.2. Predicting Rebleeding

Manifesting as externalisation of blood, tachycardia and hypotension, or a drop of more than 20 g/L after stabilisation of haemoglobin [4], rebleeding necessitates further intervention and leads to increased length of stay and associated costs. Identification of patients at high risk of rebleeding can help determine appropriate level of care and observation period.

The Forrest classification has been shown to be of prognostic value in predicting ulcer rebleeding in in multiple studies [22,23]. A meta-analysis in 2008 showed that endotherapy significantly reduced the rate of rebleeding (or persistent bleeding) in actively bleeding ulcers (risk ratio 0.29 95% CI 0.20 to 0.43) and in ulcers with a visible vessel (risk ratio 0.49, 95% CI 0.40 to 0.59) [24].

With respect to composite scores, the international multicentre prospective study by Stanley et al., reported that the PNED, which includes rebleeding as a variable, unsurprisingly performed significantly better than other scores with AUROC 0.85, compared with GBS (0.62, *p* < 0.001), RS (0.64, *p* < 0.01), pRS (0.62, *p* < 0.001) and AIMS-65 (0.60, *p* < 0.001). (7) This finding was supported by an Italian prospective multicentre cohort study including 2307 patients, which also showed that the PNED performed well (AUROC 0.87) compared to RS (0.64), ABC (0.62), AIMS-65 (0.62) and GBS (0.62, *p* < 0.00 for all) [25].

A single-centre, prospective Danish study including 831 patients comparing the performance of the GBS, an age-modified GBS, Rockall, Baylor Bleeding score (BBS) and CSMCPI showed no significant difference in performance and modest AUROCs in predicting rebleeding (AUROC 0.772, 0.767, 0.767, 0.775, 0.806, respectively) [26], with this finding supported by other studies [27,28].

The Forrest classification is a useful aid in the prediction of rebleeding in ulcers and is the basis for determining need of therapeutic intervention in recent European UGIB guidelines [4]. A weakness of the classification is that the identification of ulcer stigmata is subject to operator interpretation. Of the composite scores, with the exception of the PNED, no score was particularly discriminative at determining risk of rebleeding.

### 3.3. Predicting the Need for Endoscopic or Surgical Intervention

Pre-intervention scores can be clinically useful in predicting a need for endoscopic or surgical therapy. The GBS was designed to predict the need for in-patient intervention and performs better than other pre-intervention scores in this regard. Interestingly, a prospective study in Korea involving 1584 patients showed that the GBS performed comparably to the RS in predicting need for intervention and was significantly better than the pRS (AUROC 0.705 vs. 0.727 vs. 0.601, *p* = 0.282 and *p* < 0.0001, respectively) [29].

A recent retrospective Spanish study including 795 patients from a single-centre compared the performance of the newer risk scores, the ABC and MAP(ASH) to GBS and AIMS-65 in determining a need for intervention (a composite of red blood cell transfusion and endoscopic, radiological or surgical therapy). No significant difference in discriminative ability was found between GBS (AUROC 0.76, 95% CI 0.70–0.81) MAP(ASH) (0.75, 95% CI 0.69–0.81) and ABC (0.72 95% CI 0.66–0.77). The paper also compared the scores’ ability to specifically predict endoscopic intervention, with no score preforming well (all AUROCs <0.6) [30].

The performance of the pre-endoscopic risk scores was also reported to be suboptimal in predicting need for endoscopic intervention in a single-centre Korean study involving 523 patients, which found that only the RS score, which includes endoscopic findings, had an AUROC >0.6 (0.75 95% CI 0.71–0.79) compared to the pRS (0.52 95% CI 0.47–0.57), AIMS-65 (0.55, 95% CI 0.50–0.60) and GBS (0.59, 95% CI 0.54–0.64) [31].

Unlike the Spanish study, the prospective multicohort study performed by Marmo et al., found the GBS performed best (AUROC 0.75) in predicting need for any intervention compared to the PNED (0.69 *p* < 0.005), RS (0.69 *p* < 0.02), ABC (0.62 *p* < 0.000) and AIMS-65 (0.60 *p* < 0.000). With respect to specific intervention, it found the RS performed best (AUROC 0.69) in predicting a need of surgery or interventional radiology with no other score reaching an AUROC >0.6) [25].

Studies over the last two decades have suggested that endoscopy performed within the first 24 h of UGIB is beneficial [32,33]. Exact timing of intervention within this period is less clear. A Danish cohort study including 12,601 patient with peptic ulcer bleeding showed that endoscopy 6 to 24 h after admission to hospital was associated with lower in-hospital mortality in haemodynamically unstable patients compared with outside this time period (odds ratio 0.73, 95% CI 0.54–0.98) [34]. A subsequent RCT including 516 patients with GBS ≥12 showed no difference in 30-day mortality in those undergoing urgent (<6 h) endoscopy compared to early (6–24 h) endoscopy, with hazard ratio 1.35, 95% CI 0.72–2.54, *p* = 0.43) [35].

### 3.4. Predicting the Need for Red Blood Cell Transfusion

Multiple studies have found the GBS to be most discriminative for predicting need for blood transfusion. A United Kingdom multicentre study involving 1555 patients presenting with UGIB showed the GBS (AUROC 0.93, 95% CI 0.92–0.94) performed well in predicting a need for transfusion and was significantly more discriminative than both the pRS (0.73, 95% CI 0.70–0.76, *p* < 0.0005) and RS (0.92, 95% CI 0.90–0.94, *p* < 0.0005). In the same study, when compared directly, the RS significantly outperformed the pRS in predicting transfusion [36]. The GBS (AUROC 0.84, 95% CI 0.80–0.88) was also found to be significantly better than the pRS (0.62, 95% CI 0.57–0.67), RS (0.61, 95% CI 0.56–0.67) and AIMS-65 (0.60, 95% CI 0.55–0.65) in predicting transfusion requirement in the Korean study described above [31]. In addition, a prospective Croatian study including 1012 patient with peptic ulcer bleeding compared the GBS to pRS and BBS and found the GBS to be superior (AUROC 0.83, 95% CI 0.80–0.85 vs. 0.63, 95% CI 0.59–0.66 vs. 0.59 95% CI 0.55–0.62, respectively) [37]. 

Based on multiple comparison studies, the GBS performed consistently better than other risk scores with a good AUROC; therefore, it appears to be the most appropriate score for predicting need for blood transfusion.

## 4. Low-Risk Patients

The use of pre-endoscopic risk scores for early identification of very low-risk patient presenting with UGIB allows for a potential avoidance of admission or early discharge for these patients. Avoidance of in-patient endoscopy also confers benefits on resource utilisation and costs. From a patient safety perspective, in these scenarios, a high sensitivity is of much greater importance than high specificity.

A systematic review including 16 studies, assessing the predictive value of pre-endoscopic risk scores for 30-day serious adverse events (a composite including 30-day mortality, recurrent bleeding and need for intervention), was conducted. The review found that the GBS had the greatest sensitivity (0.98) compared to the pRS (0.93) and AIMS-65 (0.24). No score had good specificity (0.16, 0.24 and 0.61, respectively) [38].

An international observation study including 2305 patients comparing threshold of GBS and age-modified GBS found all systems had good sensitivity (97%) but a cut-off value of GBS ≤1 and ≤2 and the modified GBS systems had higher specificity compared to a cut-off GBS 0 (40–49% vs 22%, respectively *p* < 0.001) [39]. The authors found a GBS cut-off ≤2 had highest specificity; however, 3% of patients classified low-risk using this measure had adverse outcomes and s GBS ≤1 was therefore suggested as the optimal cut-off.

While a GBS 0-1 appears safe for out-patient management, given the significant cost implications of in-patient admissions, the safety of extending the GBS low risk threshold have been explored. A single-centre retrospective study of 399 patients reported that extending it to GBS 2 or 3 reduced the negative predictive value for excluding the need for endoscopic intervention to 98.53% and 98.77%, respectively. Two patients with a GBS 3 died from UGIB, with the authors concluding, in the context of non-variceal UGIB, that the GBS could be extended to ≤2 [40]. However, given the larger evidence base for the low-risk threshold to be GBS ≤ 1, the most recent iteration of Asian-Pacific, European, American and international guidelines [4,41,42,43] recommend a GBS ≤ 1 as the threshold for determining low-risk patients suitable for out-patient management. One exception may be during peak periods of a viral pandemic, when resources are stretched and endoscopy services are under severe pressure. In this situation, a Scottish multicentre prospective study has suggested that the low-risk threshold could be pragmatically (and temporarily) extended to GBS ≤ 3 [44]. The authors of the study reported a mortality rate in GBS 0-3 of 2.4% (2/84) compared with GBS > 3 15.3% (48/313). No difference in outcomes was observed between patients with GBS 0-1 and GBS 2-3.

During development of the CANUKA score, its performance was compared with the GBS and pRS using the internal validation dataset. This showed that patients with CANUKA ≤ 1 had a positive predictive value (PPV) of safe discharge of 96.3% compared to GBS ≤1 and pRS ≤ 1 (PPV 95.3% and 77.4%, respectively). However, the CANUKA ≤ 1 only applied to 7% of patients within the dataset compared to the 24% of patients with GBS ≤ 1 [12].

## 5. Predicting a Composite Endpoint

The ideal UGIB risk score would accurately predict all the outcomes discussed above, simplifying stratification for the attending clinician to a single calculation. The recent GBS and CANUKA scores were designed to predict a composite endpoint and a number of validation and comparison studies have looked to address this area. Composite endpoints often combine mortality and in-patient intervention as two important measures in higher risk patients; however, in-patient intervention itself can be defined by a combination of endpoints (endoscopy, surgical or radiological intervention, and blood transfusion), making interstudy comparisons difficult.

**Figure 2:**
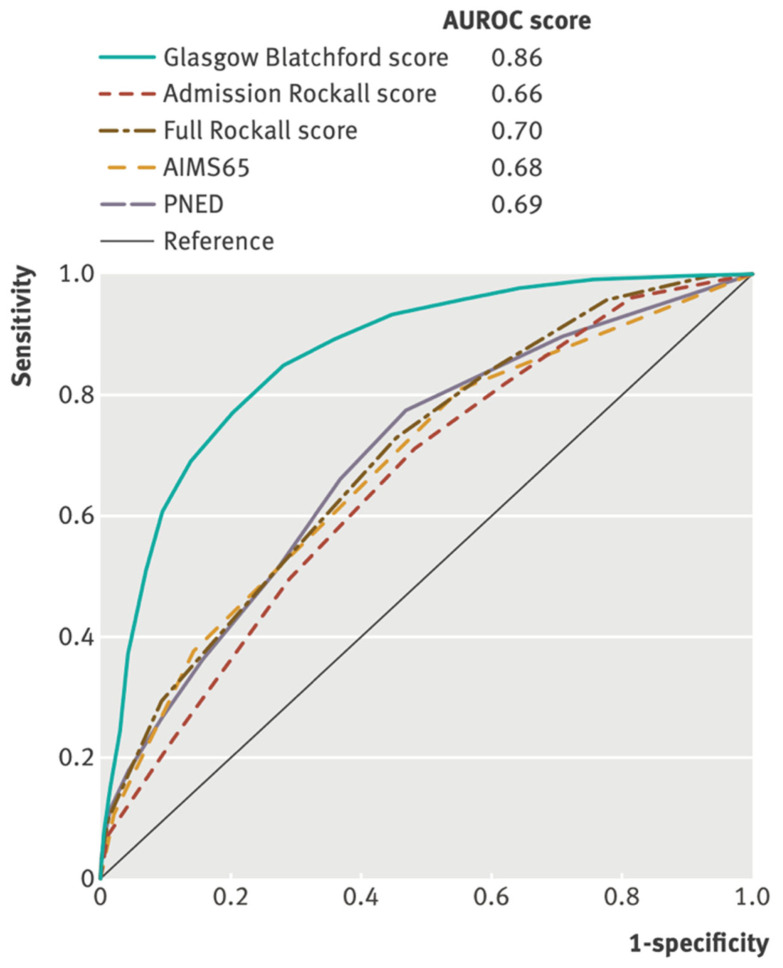
Comparisons of scores in the prediction of a need for any intervention (transfusion, endoscopic treatment, interventional radiology or surgery) or 30-day mortality *(n* = 1704). All figures compared patients with complete data for all compared scores. AUROC—area under receiver operating characteristic curve [7].

A composite endpoint of need for hospital-based intervention (blood transfusion, endoscopic treatment, interventional radiology or surgery) or death was considered by the international multicentre prospective study (Figure 2) [7]. It showed that the GBS performed best at predicting this composite endpoint (AUROC 0.86) compared to the PNED (0.69, *p* < 0.001)—a post-intervention calculated score, RS (0.70, *p* < 0.001), pRS (0.66, *p* < 0.001) and AIMS65 (0.68, *p* < 0.001).

A retrospective, cross-sectional, observation study performed in Switzerland including 1521 patients also showed the GBS (AUROC 0.77 95% CI, 0.75–0.79) and modified GBS (0.78, 95% CI, 0.76–0.81) had the highest discriminatory capacity to determine the composite end point of in-hospital death or need of intervention (blood transfusion, endoscopic treatment or surgery), outperforming the AIMS-65 (0.68, 95% CI 0.66–0.71) and pRS (0.65, 95% CI 0.62–0.68) [45].

The authors of the initial CANUKA study reported that the GBS performed better than CANUKA in predicting a combined poor outcome defined as one or more of 30-day mortality, 30-day rebleeding, surgical or radiological intervention, need for therapeutic endoscopy or blood transfusion. As described above, the GBS has been consistently shown to be the best risk score for predicting need for blood transfusion and when this variable was excluded from the composite, the CANUKA and GBS performed comparably (AUROC 0.78 95% CI 0.76–0.80 vs. 0.79 95% CI 0.76–0.81 *p* = 0.53) [12].

## 6. The Future of Risk Scores

Current UGIB risk scores are not dynamic; however, these are utilised during the admission phase of a patient’s stay. As such, they enable stratification soon after presentation but do not aid in repeated, dynamic clinical decision making throughout the admission, during which the patients’ condition can rapidly improve or deteriorate, altering their risk profile. The PNED which includes rebleeding and failure of endoscopic treatment as calculated variables has been suggested as the best risk scores for re-evaluation [25]; however, the recently described machine learning (ML) methodology may supersede this.

Automated, integrated systems that detect impending clinical deterioration in hospitalised patients have been shown to improve 30-day outcomes [46], with potential for similar systems to be utilised in patients with UGIB if embedded in electronic healthcare records. A prospective multicentre American study in 2011 highlighted the potential for such models in risk scores. Including 2380 patients with non-variceal UGIB, it showed that a pre-endoscopic computerised artificial neural network (ANN), which performed dynamic assessment, was more significantly more discriminative in predicting 30-day mortality compared to the RS (AUROC 0.95, 95% CI 0.92–0.98 vs. 0.67, 95% CI 0.65–0.69, *p* < 0.001) [47].

A more recent study included 1958 patients in the development of a gradient boosting ML model to predict a composite end point of hospital-based intervention (blood transfusion or haemostatic intervention) or death within 30 days. Subsequently validated using 399 patients, the ML model (AUROC 0.90) outperformed the GBS (0.87, *p* = 0.04), pRS (0.66, *p* < 0.001) and AIMS-65 (0.64 *p* < 0.001). The model also outperformed the GBS in the identification of low-risk patients, with a specificity of 26% at 100% sensitivity compared to a specificity of 12% for GBS 0 (sensitivity 100%) [48]. This improved specificity would enable a greater number of low-risk patients to be discharged for out-patient investigation with associated financial and resource benefit.

ML and artificial intelligence will enable more complex scoring algorithms to be used that can be embedded in electronic health care records, enabling dynamic scoring and simplifying risk stratification for the clinician.

## 7. Conclusions

UGIB risk scores are important tools that should be utilised as part of the management of UGIB to enable stratification of low- and high-risk patients.

Of the current scores, the GBS has been shown to perform most consistently in the identification of low-risk patients who can safely avoid admission. A GBS < 1 is recommended by most current UGIB guidelines as the optimal threshold to identify patients suitable for out-patient management. Given the relatively low specificity achieved at this level, some patients who could be safely discharge may be admitted. Further research is required to determine if newer scores can provide better specificity with comparable sensitivity.

For prediction of mortality, the pre-endoscopic AIMS-65 score performs reasonably well when compared with post-endoscopic scores. However, the more recent ABC score appears to be superior in predicting mortality. No score currently can accurately predict need for endoscopic therapy therefore more work is required in this regard.

ML models have shown significant promise in risk scoring for UGIB and may address the criticism that current risk scores’ complexity limits their clinical use. Though predominantly limited to the research setting at present, ML modelling use is likely to become more widespread in the near future and a valuable tool for clinicians who manage patients with UGIB.
